# College of Veterinary Surgeons

**Published:** 1871-08

**Authors:** John Busted

**Affiliations:** President of the Faculty


College of Veterinary Surgeons.—The Faculty of the N. Y. College of Veterinary Surgeons have *Resolved*, That each County Medical Society in this State, shall have the privilege of a half Free Scholarship in this Institution. Please make this known to your Society. I herewith transmit the Annual Announcement of the College, and am

Respectfully Yours,

To Prof. J. F. Miner, *Dean Buffalo Med. College.*

John Busted, M. D. *President of the Faculty.*

